# A Virtual Cardiovascular Care Program for Prevention of Heart Failure Readmissions in a Skilled Nursing Facility Population: Retrospective Analysis

**DOI:** 10.2196/29101

**Published:** 2021-06-01

**Authors:** Daniel M Friedman, Jana M Goldberg, Rebecca L Molinsky, Mark A Hanson, Adam Castaño, Syed-Samar Raza, Nodar Janas, Peter Celano, Karen Kapoor, Jina Telaraja, Maria L Torres, Nayan Jain, Jeffrey D Wessler

**Affiliations:** 1 Heartbeat Health, Inc. New York, NY United States; 2 Vagelos College of Physicians & Surgeons Columbia University New York, NY United States; 3 Division of Epidemiology & Community Health School of Public Health University of Minnesota Minneapolis, MN United States; 4 Innovative Practice & Telemedicine Section Department of Emergency Medicine The George Washington University Washington, DC United States; 5 Cassena Care, LLC Woodbury, NY United States; 6 Division of Cardiology Department of Medicine Columbia University Irving Medical Center New York, NY United States

**Keywords:** heart failure, readmissions, skilled nursing facilities, posthospitalization, cardiovascular, cardiology, outcome, cost, virtual care, telehealth, telemedicine, mobile health, mHealth, digital health

## Abstract

**Background:**

Patients with heart failure (HF) in skilled nursing facilities (SNFs) have 30-day hospital readmission rates as high as 43%. A virtual cardiovascular care program, consisting of patient selection, initial televisit, postconsultation care planning, and follow-up televisits, was developed and delivered by Heartbeat Health, Inc., a cardiovascular digital health company, to 11 SNFs (3510 beds) in New York. The impact of this program on the expected SNF 30-day HF readmission rate is unknown, particularly in the COVID-19 era.

**Objective:**

The aim of the study was to assess whether a virtual cardiovascular care program could reduce the 30-day hospital readmission rate for patients with HF discharged to SNF relative to the expected rate for this population.

**Methods:**

We performed a retrospective case review of SNF patients who received a virtual cardiology consultation between August 2020 and February 2021. Virtual cardiologists conducted 1 or more telemedicine visit via smartphone, tablet, or laptop for cardiac patients identified by a SNF care team. Postconsult care plans were communicated to SNF clinical staff. Patients included in this analysis had a preceding index admission for HF.

**Results:**

We observed lower hospital readmission among patients who received 1 or more virtual consultations compared with the expected readmission rate for both cardiac (3% vs 10%, respectively) and all-cause etiologies (18% vs 27%, respectively) in a population of 3510 patients admitted to SNF. A total of 185/3510 patients (5.27%) received virtual cardiovascular care via the Heartbeat Health program, and 40 patients met study inclusion criteria and were analyzed, with 26 (65%) requiring 1 televisit and 14 (35%) requiring more than 1. Cost savings associated with this reduction in readmissions are estimated to be as high as US $860 per patient.

**Conclusions:**

The investigation provides initial evidence for the potential effectiveness and efficiency of virtual and digitally enabled virtual cardiovascular care on 30-day hospital readmissions. Further research is warranted to optimize the use of novel virtual care programs to transform delivery of cardiovascular care to high-risk populations.

## Introduction

### Background

Heart failure (HF) is the leading cause of hospitalization and readmission in the Medicare population [1]. Among the more than 1.5 million residents within skilled nursing facilities (SNFs) in the United States, 20%-37% carry a diagnosis of HF [2]. In the current era, 1 in 4 older patients hospitalized with HF is discharged to a SNF [3]. SNFs operate on transfer agreement(s) with 1 or more participating hospitals to provide skilled nursing, medical care, and rehabilitation services for patients that are injured, disabled, or sick [4].

HF readmission rates, while high at baseline, are even higher within the SNF population. Although community HF readmission rates average 22%, 30-day HF readmission rates in the SNF setting range from 27% to 43% [3,5,6]. There is great interest in reducing this “revolving door” phenomenon within the growing SNF population, as patients are living longer with greater disease severity and multiple comorbidities [4,7]. These readmissions also come at great expense to the health care system, averaging over US $9000 for a typical HF readmission [8].

Virtual visits have been identified as a potential solution to provide access to health care populations at high risk for readmission, such as those with HF. A pilot study conducted by the Cleveland Clinic examined the feasibility and safety of substituting in-person visits with virtual visits for patients with HF transitioning from hospital to home [9]. The authors found that there were no significant differences in hospital readmissions, emergency room visits, or death between the 2 groups. The no-show rate with virtual visits also trended lower than the rate for in-person visits [10].

### The Impact and Resilience of Virtual Cardiovascular Care

The COVID-19 pandemic propelled virtual care to center stage in 2020 given the need to reduce exposure risk among both health care workers and patients, particularly in the SNF setting. For the first time ever, virtual visits surpassed in-person visits in percentage of overall visit volume. Survey data reported that virtual visits ballooned from 9% of patient interaction prepandemic to 51% at its peak [11]. Although televisits declined in subsequent months, virtual care has had significant staying power and will likely outlast the pandemic, as supported by recent Centers for Medicare and Medicaid Services (CMS) telehealth expansion [12]. In the 2021 Physician Fee Schedule, CMS has expanded telehealth coverage, reducing frequency limitations on telehealth services in nursing facilities, allowing for more frequent virtual visits to occur should they be indicated [13]. Further, the Federal Communications Commission has announced additional funding to explore Connected Care Pilot Programs [14].

There are often barriers to access to specialty cardiology care when a patient is in the SNF setting, which may be reduced by virtual care [9]. Virtual visits may eliminate overhead required for an in-person visit from a SNF setting, such as preparing the patient for transfer via ambulance or ambulette and receiving them upon their return. In-person clinic visits may expose already high-risk patients to infectious diseases, including SARS-CoV-2 and typical seasonal illnesses such as influenza. Given the susceptibility of SNF settings to outbreaks of contagious illnesses, the elimination of unnecessary exposure has the potential to benefit both residents and staff. Such visits may not only reduce readmissions, but also reduce costly emergency department visits that do not ultimately result in admission. Potential benefits to virtual cardiology care in the SNF setting are summarized in Table 1.

Despite concerns about physical examination limitations during virtual care, developing data suggest that remote assessment of jugular venous pressure may correlate with invasive right heart catheterization measurements. In one study, bedside and remote jugular venous pressure assessments were comparable and significantly correlated with invasive measurements [15]. Other modified maneuvers can be performed during a virtual examination, such as an assessment for lower extremity edema. To observe any edema, the provider may ask a patient by video to show their legs and press on them, any indentation suggesting pitting edema. If patients are in a SNF at the time of virtual visit, staff nurses may assist patients with their examination, helping them to overcome any barriers due to mobility. Further, the ability for a staff member to assist a patient with a virtual examination is invaluable given the existing disparities in the telehealth space, particularly in the Medicare or Medicaid population. These patients may have limited access to computers with an internet connection or smartphones [16].

**Table 1:** Benefits of virtual cardiology care in the SNF^a^ setting [9].

Group	Potential benefits
SNF patients	Increased access to timely care Increased access to follow-up visits Reduced disruption (ie, no transport) Reduced exposure to infectious diseases (eg, flu, SARS-CoV-2) Reduced emergency department visits or readmissions Reduced time to optimize guideline-directed medical theory
Providers	Increase care delivery to high-risk population Reduced disruption to clinic flow Increased feasibility of frequent follow-up televisits History can be supplemented by SNF nurse during televisit Physical examination can be performed by a SNF nurse during televisit
Skilled nursing facility	Reduced disruption (ie, no transport) Reduced exposure to infectious diseases (eg, flu, SARS-CoV-2) for all residents Reduced reimbursable days lost to readmission
Payers	Reduced costs (readmission, transportation)
Health care system	Reduced costs Support research efforts

^a^SNF: skilled nursing facility.

### Purpose

Recent paradigm shifts in policies and attitudes toward virtual care have opened the door for new methods of care delivery, particularly in high-risk populations. While both specialty and virtual care delivery have been points of interest for posthospitalization populations, access challenges, including technology literacy and familiarity, have prevented widespread adoption of such approaches. Introduction of virtual cardiovascular care into the SNF setting offers a compelling opportunity to address these challenges. Given the increase in complex, older patients living with HF transitioning from hospitals to SNFs, this population may stand to benefit from novel, digitally enabled care pathways.

The purpose of this study was to assess the feasibility, efficacy, and efficiency of virtual cardiovascular care delivery on a population of patients with a preceding index admission for HF in a SNF setting. We hypothesized that a virtual cardiovascular care program involving at least one virtual consultation would reduce the 30-day readmission rate for patients with HF relative to the expected rate for this population for reasons including the following: (1) direct communication of a care plan between the patient and specialist, (2) specialist inference of gaps in the patient’s medical history from the visit and chart review, and (3) titration of pharmacotherapies. Relatedly, the introduction of virtual care within the controlled and assisted environment of a SNF could help to bridge the technology literacy and familiarity divide that is associated with the study demographic, in addition to saving costs attributed to prevented hospital readmissions. This investigation precedes an expected larger study of payer-referred postacute patients along with a future randomized control trial targeting similar high cardiovascular risk patients.

## Methods

### Study Overview

This study represents an uncontrolled initial exploration conducted over 7 months across a population of patients receiving long-term care and subacute rehabilitation in 11 SNFs and 3510 beds in the New York metropolitan area. A total of 185 patients were evaluated based on their referral by the SNF staff. The SNF care team identified patients with a diagnosis of congestive HF and initiated program enrollment by scheduling a virtual consultation (consult) with a cardiologist on the Heartbeat Health software platform. Following enrollment and participation in a virtual cardiovascular care program, retrospective chart reviews were conducted across all study participants and results were tabulated with a specific focus on hospital readmissions and related outcome improvements and estimated cost savings.

### Virtual Cardiovascular Care Technology Platform

Virtual cardiovascular care was enabled by a software technology platform provided by Heartbeat Health, a digital health company with a focus on virtual cardiovascular care delivery. The platform consists of a software provider interface and patient facilitator interface. The interfaces are linked by a secure cloud-based infrastructure connecting the experiences. Both of the experiences, provider and patient, can be delivered over mobile, tablet, or laptop device viewports. The technology allows for patient enrollment, patient or patient-assisted requests for care, provider review of patient records, instantaneous virtual video and voice visits, and ongoing patient–provider coordination, among other capabilities. The platform can be used in conjunction with electronic medical record of the SNF or practice. The technology was deployed in a provider setting with a group of cardiologists overseeing care and in a SNF setting with the clinical staff, including nurses and nurse managers, assisting SNF patients in the delivery of care.

### Virtual Cardiovascular Care Program

**Figure 1:**
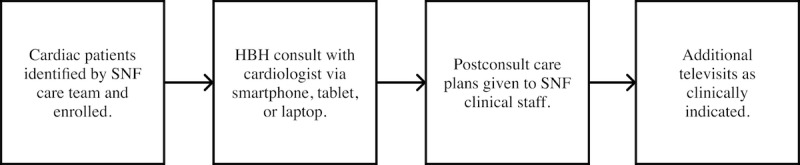
A flowchart of the virtual cardiovascular care program for patients. HBH: Heartbeat Health; SNF: skilled nursing facility.

Virtual cardiovascular care was made available to patients with a variety of cardiac conditions, though only patients with HF were included in this study. An initial online request for virtual cardiovascular care was submitted to Heartbeat Health by a representative from the SNF. Pertinent cardiac symptoms and diagnoses were noted in the request alongside any additional notable context. The first consult was scheduled within 1-2 business days of this request. Consults were performed by a remote cardiologist with a SNF nurse at the patient’s bedside. Postconsult care plans were provided to the SNF clinical staff for implementation. Some patients required additional consults that were scheduled by the cardiologist as clinically indicated. Consults were typically focused on symptom assessment, volume management, and maximization of guideline-directed medical therapy (GDMT). Heartbeat Health and the cardiology consult team were not involved in the decision to send patients to the hospital for readmission; SNF clinical teams made this decision autonomously on an as-needed basis (Figure 1).

We performed a retrospective case review of cardiovascular consultations that occurred between August 2020 and February 2021. Data were deidentified and aggregated for analysis. Inclusion criteria included an index hospitalization for HF, either HF with reduced ejection fraction (HFrEF) or HF with preserved ejection fraction (HFpEF), an initial virtual consultation within 30 days of arrival to the SNF, and not receiving a comfort care protocol. Patients who were discharged home from the SNF setting within 30 days of arrival were considered lost to follow-up, as patient readmission status was determined using SNF data after 30 days from the date of admission.

**Figure 2:**
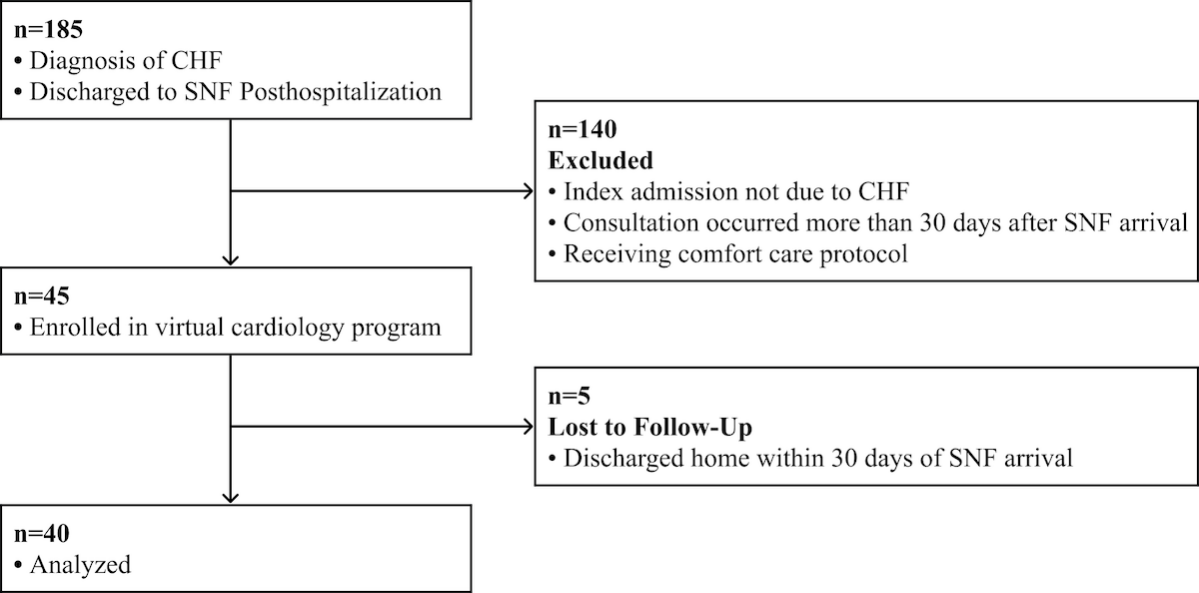
Patient flow through the analysis. CHF: congestive heart failure, SNF: skilled nursing facility.

In a population of 3510 SNF beds, 185 patients received virtual cardiology care via the Heartbeat Health program. A total of 45 patients met inclusion criteria, of which 40 were analyzed and 5 were lost to follow-up, as they were discharged from SNF to home within 30 days of their hospitalization (Figure 2).

### Statistical Analysis

All analyses were conducted using R (version 4.0.3; R Foundation for Statistical Analysis). Participant baseline characteristics are presented as percentages or means (SD). A retrospective analysis was conducted comparing readmission status among participants. Percentages or means (SE) were computed across readmission status. *P* values for difference among readmission status were obtained from Fisher exact test or analysis of variance tests. Hedges *g* (a corrected version of Cohen *d* for smaller sample sizes) and 95% CIs were computed to assess effect size for continuous risk factors. Lastly, odds ratios and 95% CIs across readmission status are presented for categorical risk factors.

## Results

At baseline, patients were on average 80.5 (SD 10.4) years old, 50% (20/40) were female, 65% (26/40) White, 28% (11/40) Black, 5% (2/40) Hispanic, and 3% (1/40) Asian (Table 2). In accordance with recent literature, race should be viewed as a proxy metric for social, environmental, and structural factors rather than as a biological risk factor [17]. Baseline comorbidities were typical of an older SNF population with HF and included coronary artery disease (17/40, 43%), hypertension (38/40, 95%), diabetes (10/40, 25%), and a history of stroke (4/40, 10%). Mean ejection fraction was 41.6% (SD 17.9), with approximately 40% (16/40) of patients having HFpEF, 53% (21/40) having HFrEF, and the remaining 8% (3/40) having unspecified HF. Patients in New York Heart Association (NYHA) Class II or Class III were classified together and represented 93% (37/40) of patients. No patients were NYHA Class I, and 8% (3/40) were NYHA Class IV.

**Table 2:** Baseline characteristics.

Characteristic			Values (N=40)
Age (years), mean (SD)			80.5 (10.4)
Ejection fraction (%), mean (SD)			41.6 (17.9)
Systolic blood pressure (mmHg), mean (SD)			127.5 (19.4)
Diastolic blood pressure (mmHg), mean (SD)			67.7 (9.6)
**Sex, n (%)**			
	Male	20 (50)	
	Female	20 (50)	
**Race, n (%)**			
	Black	11 (28)	
	Hispanic	2 (5)	
	Asian	1 (3)	
	White	26 (65)	
**HF^a^ type, n (%)**			
	HF with reduced ejection fraction	21 (53)	
	HF with preserved ejection fraction	16 (40)	
	Unknown	3 (8)	
Coronary artery disease, n (%)			17 (43)
Stroke, n (%)			4 (10)
Hypertension, n (%)			38 (95)
Diabetes, n (%)			10 (25)
Chronic kidney disease, n (%)			15 (38)
Serum creatinine, mean (SD)			1.64 (1)
Chronic obstructive pulmonary disease, n (%)			5 (13)
**New York Heart Association Class, n (%)**			
	I	0 (0)	
	II-III	37 (93)	
	IV	3 (8)	
ACEI^b^, ARB^c^, or ARNI^d^, n (%)			11 (28)
Beta blocker, n (%)			28 (70)
Loop diuretic, n (%)			36 (90)
Aldosterone inhibitor, n (%)			7 (18)
Intravenous inotrope, n (%)			3 (8)

^a^HF: heart failure.

^b^ACEI: angiotensin-converting enzyme inhibitor.

^c^ARB: angiotensin receptor blocker.

^d^ARNI: angiotensin receptor-neprilysin inhibitor.

**Figure 3:**
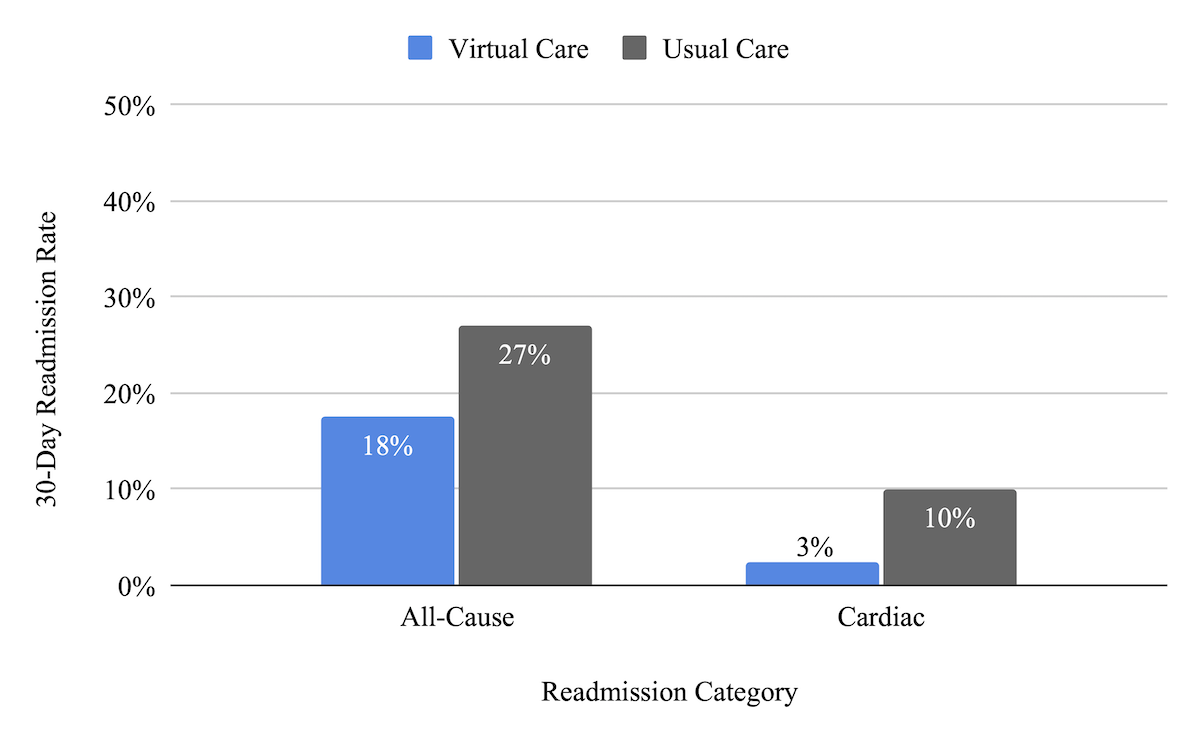
Readmission rate by cause [3,15].

Of the 40 patients analyzed, 1 patient (3%) was readmitted for a cardiac cause, as compared with the usual care readmission rate of 10% for this population (Figure 3) [18]. Seven patients (18%) had all-cause readmissions (inclusive of the 1 cardiac readmission), as compared with the usual care readmission rate of 27% for this population [3].

**Figure 4:**
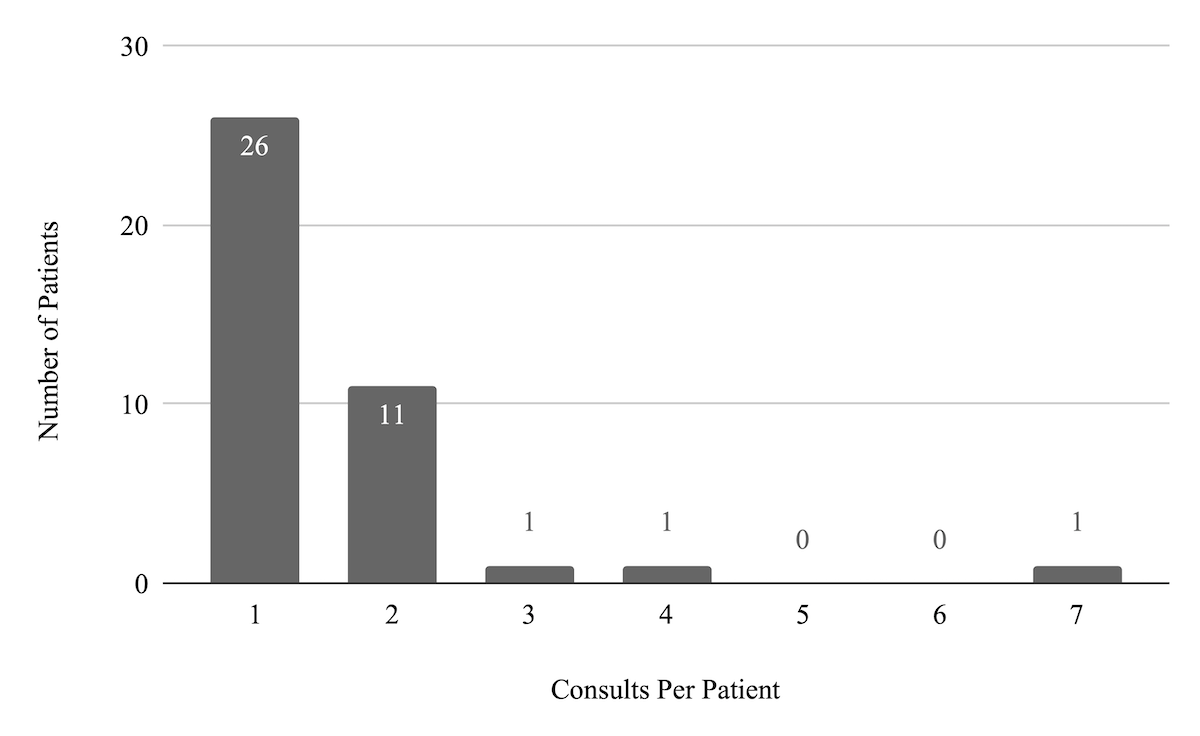
Number of virtual consults per patient.

A total of 26/40 patients (65%) required only 1 virtual consultation, whereas 14/40 (35%) required more than 1 consultation as requested by SNF medical staff or cardiology discretion (Figure 4). One patient required 7 consults during the 30-day period. Additional consults were most commonly called for volume management followed by blood pressure control.

Retrospective associations between readmission status and patient characteristics are shown in Table 3. Significant differences were found between those readmitted and those not readmitted with respect to age (*P*=.04), race (*P*=.04), and number of consults (*P*=.006). Mean age (SE) among those readmitted was significantly lower compared with those who were not readmitted (73 [4.3] vs 82 [1.7], *P*=.04). Readmission status also significantly differed by race (*P*=.04). Specifically, when comparing readmission status among Black and White patients, the odds of readmission among the former was 9.21 times the odds of readmission among the latter (*P*=.01, 95% CI 1.17-119.50). Lastly, the mean number of consults among those readmitted was significantly higher compared with those who were not readmitted (*P*=.01).

Of the patients readmitted, the majority were diagnosed with HFrEF (5/7, 71%) and had at least two consults (5/7, 71%), though each patient had a unique reason for readmission (Table 4).

**Table 3:** Retrospective associations between hospital readmission and characteristics of participants (n=40).

Characteristic			Not readmitted (n=33)		Readmitted (n=7)		*P* value		Hedges *g* or odds ratio (95% CI)^a,b^
Age (in years), mean (SE)			82.0 (1.7)		73.4 (4.3)		.04		0.84 (0 to 1.68)
Ejection fraction (in %), mean (SE)			42.9 (3.4)		36.4 (6.8)		.40		—^c^
Systolic blood pressure (in mmHg), mean (SE)			129.3 (3.4)		119.1 (6.7)		.21		—
Diastolic blood pressure (in mmHg), mean (SE)			68.2 (1.7)		65.0 (3.2)		.42		—
Number of consults, mean (SE)			1.33 (0.11)		2.57 (0.78)		.006		–1.2 (–2.06 to –0.34)
Days until first consult, mean (SE)			10.00 (1.4)		5.29 (1.44)		.15		—
Number of comorbidities, mean (SE)			2.18 (0.17)		2.43 (0.57)		.58		—
Number of HF^d^-related medication classes, mean (SE)			3.12 (0.17)		3.14 (0.26)		.95		—
**Race (All), n (%)**							.04		—
	White	24 (73)		2 (29)					
	Black	6 (18)		5 (71)					
	Hispanic	2 (6)		0 (0)					
	Asian	1 (3)		0 (0)					
**Race (White and Black only), n (%)**							.01		9.21 (1.17 to 119.50)
	White	24 (80)		2 (29)					
	Black	6 (20)		5 (71)					
**Sex, n (%)**							>.99		—
	Female	17 (52)		3 (43)					
	Male	16 (48)		4 (57)					
**Type of HF, n (%)**							.82		—
	Unknown	3 (9)		0 (0)					
	HF with preserved ejection fraction	14 (42)		2 (29)					
	HF with reduced ejection fraction	16 (48)		5 (71)					
Chronic kidney disease, n (%)			11 (33)		4 (57)		.39		—
Chronic obstructive pulmonary disease, n (%)			4 (12)		1 (14)		>.99		—
Diabetes mellitus, n (%)			9 (27)		1 (14)		.65		—
Hypertension, n (%)			32 (97)		6 (86)		.32		—
Coronary artery disease, n (%)			13 (39)		4 (57)		.43		—
Stroke, n (%)			3 (9)		1 (14)		.55		—
ACEI^e^, ARB^f^, or ARNI^g^, n (%)			11 (33)		0 (0)		.15		—
Beta blocker, n (%)			25 (76)		3 (43)		.16		—
Loop diuretic, n (%)			29 (88)		7 (100)		>.99		—
Aldosterone receptor antagonist, n (%)			4 (12)		3 (43)		.08		—
Intravenous inotrope, n (%)			1 (3)		2 (29)		.07		—

^a^Unpaired *t* tests and Hedges *g* test were performed for continuous outcomes along with 95% CI.

^b^Fisher exact test and odds ratios were performed for categorical variables along with 95% CI.

^c^—: Not available

^d^HF: heart failure.

^e^ACEI: angiotensin-converting enzyme inhibitor.

^f^ARB: angiotensin receptor blocker.

^g^ARNI: angiotensin receptor-neprilysin inhibitor.

**Table 4:** Characteristics of patients readmitted within 30 days.

Readmission category	Readmission reason	Days in skilled nursing facility before readmission, n	Total consults, n
Cardiac	Volume overload	15	2
Noncardiac	Pneumonia (unspecified)	11	1
Noncardiac	Pneumonia (COVID-19)	7	2
Noncardiac	Fever, hypoxia	22	2
Noncardiac	Pleural effusion	30	3
Noncardiac	Acute kidney injury	20	7
Noncardiac	Mechanical fall	26	1

## Discussion

### Principal Findings

The results of this study suggest that patients with HF who are discharged to SNFs and receive at least one virtual cardiology consultation within 30 days may have lower cardiac-related and all-cause 30-day readmission rates than the expected rates for this population.

There may be several reasons for the reduction in cardiac readmissions as evidenced by clinical literature exploring cardiovascular care in SNF settings. There may be a lack of familiarity among SNF clinical teams with current HF guidelines, which are complex and regularly evolving [5,19]. HF care plans are synthesized from data surrounding most recent left ventricular function assessment, weight trends, and parameters for uptitration of GDMT. However, measurement of weight may be a particular barrier as many SNFs have a standard protocol to weigh patients weekly, which is discordant with the daily weight trending required for many patients with HF [20]. Further, regular SNF diets may be high in sodium (>2000 mg per day), which can add to sodium retention if an order for a low-sodium diet is not clear [5]. SNFs may have limited cardiac records accessible to them upon transfer. While admission documentation may typically include physical and social information pertinent to rehabilitation, HF details and guidance on management may be scant or absent [5].

Specialty cardiologist supervision, delivered virtually, can provide a backstop of care to support HF protocols in SNF settings. The most obvious benefit of such supervision relates to the increase in access of patients to a care specialty at the time of need. Often, cardiovascular care in the SNF setting is delayed beyond advisable timeframes, resulting in less desirable outcomes and even heightened readmission risk. This guidance, critical for a successful transition of care, can be provided through a virtual cardiology consultation when otherwise unavailable from transfer documents. Access to a cardiologist who is well-versed with the necessity for uptitration of GDMT, as well as volume management, is irreplaceable in high-risk patients. Although most patients in this study only required 1 virtual consultation, repeat visits, often needed for GDMT titration, were made significantly more feasible given their virtual nature. The frequent touchpoints may have been a contributing factor to the reduced readmission rate observed in this analysis.

There were some notable differences in characteristics of patients that were readmitted to the hospital compared with those who were not. There were significantly more consultations performed on patients who were readmitted, which may be indicative of the level of acuity among these patients. While the additional visits may result in a slightly higher cost of care for these patients, the approach is compatible with GDMT and holds further outcome and cost benefits to HF population management through a reduction in readmissions. There were no significant differences in readmissions between patients with HFrEF and those with HFpEF, which is consistent with prior studies that have found similar rates of readmission between both HFrEF and HFpEF [21]. Comorbidities were also similar between those readmitted and those who were not. In terms of medical therapy, patients on intravenous inotropes trended toward readmission, though it did not reach statistical significance.

Notably, there was a significant difference between White and Black patients, with Black patients significantly more likely to be readmitted (odds ratio 9.21, 95% CI 1.17-119.50). This disparity has been identified in previous research examining HF readmissions in a large municipal health system, with Black patients having a higher odds ratio for 90-day readmissions than White patients (odds ratio 1.21, 95% CI 1.01-1.47) [22]. Given the known racial disparities in health care, particularly those facing the Black community, more research should be performed to identify the role for virtual care in actively closing existing care gaps and combating institutional racism [17].

### Limitations

This study presents an initial investigation into the relationship between virtual cardiovascular care delivery in SNF settings and overall readmissions, and subsequent work will address several known limitations to these findings. First, we compared our readmission rates with the expected readmission rates for the greater SNF population rather than baseline readmission data from the specific nursing facilities evaluated, which would have been preferred as a comparison. Second, patients who were discharged from the SNF within their 30-day readmission window were lost to follow-up. Because we were unable to capture readmission data from this group, we excluded them from analysis, introducing selection bias. Although a discharge to a private home is likely a favorable prognostic indicator, the 30-day readmission status of these patients who underwent the virtual cardiology program remains unknown. Third, the strength of this study was limited by its small sample size. In the future, we hope to replicate this study as a randomized control trial with a larger population of SNF patients, which will address several limitations including selection bias, confounding, generalizability, and consistency.

### Efficiency Gains of Virtual Cardiovascular Care

There are significant implications to consider with regard to cost and time when one replaces an ambulatory in-person visit from a SNF with a virtual one. Based on current CMS transitional care guidelines, an in-person visit is required within the first 7-14 calendar days of discharge (CPT 99495) [23]. Traditional in-person appointments come at great expense to the long-term care facility and, in turn, the health care system. In-person visits come with direct costs (eg, ambulance- or ambulette-based transportation service, accompanying transport staff) and indirect costs (eg, time spent by nurses to prepare a patient for transfer). The average scheduled visit in this study was 15 minutes in length with no necessary transportation cost. In the traditional setting, the ambulance trip from an in-network transport firm averages about US $400 (thus US $800 roundtrip), and the visit itself represents some US $200 in cost, for a total of US $1000. This is 5 times more than when an in-person visit is substituted virtually [24,25]. Readmission is the most expensive avoidable outcome, with an average cost of US $9051 per HF readmission [8]. The decreased all-cause readmission rate of 17.5% for the virtual care group in this study represents an average expected cost reduction of US $860 per patient (Table 5).

**Table 5:** Readmission rates of patients with heart failure from skilled nursing facility with virtual cardiology program versus usual care [3,8,18].^a^

Readmissions impacts	All cause (30 days)			Cardiac (30 days)	
	Usual care	Virtual care	Usual care		Virtual care
Skilled nursing facility heart failure readmission rate, %	27	17.5	10		2.5
Expected readmission cost per patient (US $)	2444	1584	905		226
Expected readmission savings per patient (US $)	N/A^b^	860	N/A		679

^a^Data surrounding average congestive heart failure readmission costs specifically due to cardiac etiologies were unavailable and thus assumed to be comparable with those of all-cause etiologies.

^b^N/A: Not applicable.

### The Opportunities for Virtual Cardiovascular Care

Virtual cardiovascular care is still in a nascent state and opportunities for its extension are numerous. The program could be expanded to include additional visits as needed from a generalist, with more of a focus on reducing all-cause readmissions. Further, more rigid protocols could be established for repeat virtual consults, with patients clinically stratified based on their readmission risk; higher-risk stratifications would warrant more frequent virtual consults as supported by GDMT protocols.

Future programs could enforce an initial cardiology consult within 7 days of SNF arrival. In this study, initial consults occurred on average 9 days after SNF arrival. Early postdischarge follow-up for patients is strongly associated with lower 30-day readmission. For instance, observational data examining administrative claims from hospitals of fee-for-service Medicare beneficiaries found that hospitals which had the lowest percentage of 1-week postdischarge follow-up rates after HF admissions had the highest 30-day readmission rates [26]. Another study investigated the postdischarge follow-up characteristics associated with 30-day readmission in patients with HF [27]. Researchers found that 50% of patients had clinical contact within 7 days (84% of the contacts were done via in-person clinic visit versus 16% telephone calls). Patients who were followed up within 7 days postdischarge had a 19% lower adjusted odds ratio of readmission.

While televisits appear beneficial in the SNF setting, virtual care resources may also bring valuable structure to the home setting upon discharge, particularly in patients with HF. Weintraub et al [28] demonstrated that pairing remote patient monitoring (measuring heart rate, blood pressure, and weight) with an HF disease management program resulted in lower HF hospitalizations when compared with standard care.

### Conclusions

The implementation of a virtual cardiovascular care program represents a promising way to reduce readmission rates in patients with HF in the SNF setting. Our findings and the discussion above should serve as a call to action for more research efforts examining postdischarge HF workflows within the virtual care space, particularly to challenge in-person requirements for transitional care management. Further research is warranted to determine how virtual care programs may not only provide additive benefit to existing care modalities, but also transform how care is delivered to improve outcomes, cost efficiency, and the overall care experience.

## Abbreviations

ACEI: angiotensin-converting enzyme inhibitor
ARB: angiotensin receptor blocker
ARNI: angiotensin receptor-neprilysin inhibitor
GDMT: guideline-directed medical therapy
HF: heart failure
HFpEF: heart failure with preserved ejection fraction
HFrEF: heart failure with reduced ejection fraction
NYHA: New York Heart Association
SNF: skilled nursing facility
